# Anti‐inflammatory drugs as risk modifiers for Alzheimer’s disease: a step towards precision prevention

**DOI:** 10.1002/alz70859_106694

**Published:** 2025-12-26

**Authors:** Helena Cortes‐Flores, Georgina Torrandell‐Haro, Roberta Diaz Brinton

**Affiliations:** ^1^ University of Arizona, Tucson, AZ USA

## Abstract

**Background:**

Inflammation is a critical factor in the onset and progression of neurodegenerative diseases, including Alzheimer’s Disease (AD). Notably, inflammatory processes are evident in AD as early as the prodromal stages. Despite this, the role of anti‐inflammatory treatments (aIT) in modifying AD risk remains unclear. This study aimed to evaluate the impact of aIT on AD incidence and to characterize patients who subsequently developed AD after drug exposure (non‐responders) and those who did not (responders).

**Method:**

A retrospective analysis was conducted using an insurance claims dataset, including patients 60 years and older. The anti‐inflammatory drugs included in the study were oral corticosteroids, non‐selective COX‐1 and 2 inhibitors, and COX‐2 selective inhibitors. Propensity score matching was applied to minimize bias by adjusting for age, gender, Charlson Comorbidity Index (CCI), and comorbidities. The final cohort included 225,742 patients, who were surveyed for a diagnosis of AD at least 1 year after their first prescription for an oral anti‐inflammatory drug, independently of their inflammation‐related medical condition. The analysis assessed different aIT classes, treatment durations, and a responder analysis to identify patient characteristics associated with AD risk. Characterization of patients included demographic characteristics, comorbidity burden, and co‐treatment profiles.

**Result:**

The propensity score matched group included 112,871 patients exposed to aIT and 112,871 patients with no exposure to any aIT. aIT use was associated with a significant decrease in the risk of AD diagnosis (RR [95%CI]: 0.35 [0.33–0.37]; P<.001), driven by a significantly reduced risk in patients undergoing aIT for at least a year. There were no statistically significant differences in AD risk reduction when comparing women to men. Patients aged 75 to 79 years and those with longer aIT exposure (6 years or longer) exhibited the greatest risk reduction. COX inhibitors were associated with superior AD risk reduction compared to corticosteroids.

**Conclusion:**

This study supports the association between aIT and a reduced risk of AD. Key factors influencing this relationship included patient age, treatment duration, and the drug class used. These nuanced findings can guide future interventions targeting neuroinflammation in AD prevention.

